# Pharmacological inhibition of dynamin‐related protein 1 attenuates skeletal muscle insulin resistance in obesity

**DOI:** 10.14814/phy2.14808

**Published:** 2021-04-27

**Authors:** Benjamin A. Kugler, Wenqian Deng, Abigail L. Duguay, Jessica P. Garcia, Meaghan C. Anderson, Paul D. Nguyen, Joseph A. Houmard, Kai Zou

**Affiliations:** ^1^ Department of Exercise and Health Sciences College of Nursing and Health Sciences University of Massachusetts Boston Boston MA USA; ^2^ School of Sports Medicine and Health Chengdu Sport Institute Chengdu China; ^3^ Department of Kinesiology East Carolina University Greenville NC USA; ^4^ Human Performance Laboratory East Carolina University Greenville NC USA

**Keywords:** insulin resistance, mitochondrial dynamics, obesity, skeletal muscle

## Abstract

Dynamin‐related protein‐1 (Drp1) is a key regulator in mitochondrial fission. Excessive Drp1‐mediated mitochondrial fission in skeletal muscle under the obese condition is associated with impaired insulin action. However, it remains unknown whether pharmacological inhibition of Drp1, using the Drp1‐specific inhibitor Mitochondrial Division Inhibitor 1 (Mdivi‐1), is effective in alleviating skeletal muscle insulin resistance and improving whole‐body metabolic health under the obese and insulin‐resistant condition. We subjected C57BL/6J mice to a high‐fat diet (HFD) or low‐fat diet (LFD) for 5‐weeks. HFD‐fed mice received Mdivi‐1 or saline injections for the last week of the diet intervention. Additionally, myotubes derived from obese insulin‐resistant humans were treated with Mdivi‐1 or saline for 12 h. We measured glucose area under the curve (AUC) from a glucose tolerance test (GTT), skeletal muscle insulin action, mitochondrial dynamics, respiration, and H_2_O_2_ content. We found that Mdivi‐1 attenuated impairments in skeletal muscle insulin signaling and blood glucose AUC from a GTT induced by HFD feeding (*p* < 0.05). H_2_O_2_ content was elevated in skeletal muscle from the HFD group (vs. LFD, *p* < 0.05), but was reduced with Mdivi‐1 treatment, which may partially explain the improvement in skeletal muscle insulin action. Similarly, Mdivi‐1 enhanced the mitochondrial network structure, reduced reactive oxygen species, and improved insulin action in myotubes from obese humans (vs. saline, *p* < 0.05). In conclusion, inhibiting Drp1 with short‐term Mdivi‐1 administration attenuates the impairment in skeletal muscle insulin signaling and improves whole‐body glucose tolerance in the setting of obesity‐induced insulin resistance. Targeting Drp1 may be a viable approach to treat obesity‐induced insulin resistance.

## Graphical abstract

We found that inhibiting Drp1 attenuates the impairment in skeletal muscle insulin signaling and improves whole‐body glucose tolerance in the setting of obesity‐induced insulin resistance. Targeting Drp1 may be a viable approach to treat obesity‐induced insulin resistance.

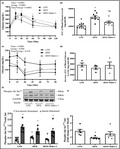

## 1 INTRODUCTION

Obesity is accompanied by a myriad of metabolic aberrations that contribute to the increased risk of type 2 diabetes (T2D; Eckel et al., 2011). One of the characteristic features of obesity and a forerunner of T2D is the reduced insulin sensitivity in skeletal muscle (Mendrick et al., 2018). Although considerable efforts have been made in understanding skeletal muscle insulin resistance, the underlying mechanisms remain incompletely understood.

Mitochondria are highly dynamic organelles that continually fuse (fusion) and divide (fission; Ferree & Shirihai, 2012). The orchestrated balance of fusion and fission is crucial in maintaining mitochondrial morphology, quality, and function (Wai & Langer, 2016). Recently, dysregulated mitochondrial dynamics have emerged as a potential cause of skeletal muscle insulin resistance (Fealy et al., 2018; Jheng et al., 2012; Nunnari & Suomalainen, 2012; Pereira et al., 2017). It has been reported that the balance of mitochondrial dynamics is shifted toward mitochondrial fission resulting in fragmented mitochondrial networks in the setting of obesity‐induced insulin resistance (Gundersen et al., 2020; Kristensen et al., 2018). Dynamin‐related protein 1 (Drp1) is a key regulator of mitochondrial fission (Tilokani et al., 2018). While Drp1‐mediated mitochondrial fission is essential in maintaining mitochondrial function and overall skeletal muscle health (Dulac et al., 2020; Favaro et al., 2019), excessive activation/upregulation of Drp1 leads to aberrant mitochondrial fission causing imbalanced mitochondrial dynamics and mitochondrial fragmentation (Giovarelli et al., 2020; Touvier et al., 2015). We and others recently reported that Drp1 is hyperactivated in skeletal muscle from obese insulin‐resistant humans, and this hyperactivation is strongly correlated with impaired skeletal muscle insulin signaling and glucose metabolism (Fealy et al., 2014; Kugler et al., 2020). Additionally, Jheng et al. demonstrated that a Drp1 knockdown provided protection from palmitate‐induced mitochondrial fragmentation and insulin resistance in the C_2_C_12_ cell culture model (Jheng et al., 2012).

Mitochondrial fission inhibitor‐1 (Mdivi‐1) is the first and by far the most accessible pharmacologic inhibitor of the mitochondrial fission protein Drp1 (Cassidy‐Stone et al., 2008b; Smith & Gallo, 2017). Mdivi‐1 has been shown to target Drp1 in mammalian cells by binding at an allosteric site and suppressing Drp1 translocation to the mitochondria as well as self‐assembly into ring‐like structures around the mitochondria (i.e., mitochondrial fragmentation; Cassidy‐Stone et al., 2008b). The therapeutic potential of Mdivi‐1 has been extensively reported in animal disease models such as Parkinson's disease, Alzheimer's disease, and various kidney diseases (Rosdah et al., 2016). In the context of insulin resistance, although one study found Mdivi‐1 transiently enhanced skeletal muscle insulin signaling in a genetic model of obese mice (Jheng et al., 2012), the efficacy of prolonged administration of Mdivi‐1 as a pharmacological approach to inhibit Drp1 and improve skeletal muscle insulin sensitivity in obesity and insulin resistance has not been fully investigated.

Therefore, the purpose of this study was to examine the efficacy of pharmacological inhibition of Drp1 in improving skeletal muscle mitochondrial dynamics and function, insulin sensitivity, and whole‐body glucose metabolism in an obesity‐induced insulin‐resistant condition. We hypothesized that obesity results in imbalanced skeletal muscle mitochondrial dynamics toward pro‐fission, mitochondrial dysfunction, impaired insulin sensitivity, and whole‐body glucose intolerance, and these negative consequences will be alleviated by pharmacological inhibition of Drp1 using Mdivi‐1.

## 2 METHODS

### 2.1 Animals and interventions

Male C57BL/6J mice were purchased (Jackson Laboratories) at 5‐weeks of age. Animals were housed in a temperature and humidity‐controlled environment and maintained on a12:12 h light–dark cycle with food and water provided ad libitum. Mice were acclimatized to the animal facility for 1‐week before any experiment.

After being acclimatized to the animal facility, mice were randomly divided into either a high‐fat diet (HFD, 45% kcal by fat, D12451, Research Diets) or a low‐fat diet group (LFD, 10% kcal by fat, D12450J, Research Diets) for 5‐weeks. After 4‐weeks of diet intervention, half of the mice in the HFD group received an intraperitoneal injection of Mdivi‐1 (20 mg/kg body weight, Enzo Life Science), whereas the other half received saline (1% dimethylsulphoxide [DMSO] in PBS) every other day for the last week of the diet intervention. Due to the poor aqueous solubility of Mdivi‐1, each dose was gently sonicated in order to produce a homogenous suspension and delivered immediately through intraperitoneal injection as previously described (Rappold et al., 2014). This dose of Mdivi‐1 has been previously reported to be safe and effective in mice (Cui et al., 2016; Rappold et al., 2014). These interventions created three experimental groups: LFD, HFD, and HFD+Mdivi‐1 (*n* = 9/group). In addition, another set of mice (*n* = 6/group) was used for insulin tolerance test (ITT) and insulin signaling measurement. All experimental procedures were approved by the Institutional Animal Care and Use Committee of the University of Massachusetts Boston.

### 2.2 Human skeletal muscle cell culture

Previously isolated human skeletal muscle cells (HSkMCs) from severely obese, insulin‐resistant women (BMI ≥40 kg/m^2^, HOMA‐IR ≥2.5, *n* = 6) and lean insulin‐sensitive women (BMI < 25 kg/m^2^, HOMA‐IR < 2.5, *n* = 6) were used in this study (Kugler et al., 2020). In brief, human skeletal muscle cells (Passage 3) were thawed, pooled together, and grown in a humidified environment with 5% CO_2_ at 37°C on a type‐I collagen‐coated flask (Greiner Bio‐one). At a confluence of ~80%–90%, myoblasts were subcultured onto type I collagen‐coated plates (Corning), 35 mm collagen‐coated glass‐bottom dish (MatTek), Seahorse XFp cell culture miniplate (Agilent Technologies), or 96 well clear bottom black polystyrene microplate (Corning) depending on experimental purposes. Upon reaching ~80%–90% confluency, myoblasts were switched to low‐serum (2% horse serum) media to induce differentiation into myotubes. On day 6 of differentiation, myotubes were treated with either Mdivi‐1 (20 μM) or vehicle (1% DMSO in PBS) for 12 h. For insulin signaling and glucose uptake measurements, myotubes were serum‐starved for 3 h, incubated with or without 100 nM of insulin for 10 min, and cell lysates were collected for further analysis as previously described (Bikman et al., 2010). All experimental procedures were repeated in three independent experiments.

### 2.3 Glucose tolerance test and insulin tolerance test

Intraperitoneal glucose tolerance test (GTT) was performed 24 h after the second to last injection of Mdivi‐1. Similarly, in another set of animals, an insulin tolerance test (ITT) was performed 24 h after the second to last injection of Mdivi‐1. Prior to GTT and ITT, mice were fasted for 6 h and given an intraperitoneal injection of either glucose (2 mg/kg body weight) or insulin (1 U/kg body weight). Blood samples were collected from the tail vein and assessed for glucose concentration before injection (0 min) and 30, 60, and 120 min post‐injection using a Contour next blood glucometer (Ascensia Diabetes Care). The area under the curve (AUC) and inverse area under the curve were calculated using the trapezoid rule during the GTT and ITT, respectively (Nagy & Einwallner, 2018).

### 2.4 Tissue collection

Twenty‐four hours after the final Mdivi‐1 injection, mice were killed using CO_2_ asphyxiation/cervical dislocation. Blood was collected immediately via vena cava puncture, centrifuged for 15 min at 2500 rpm at 4°C to collect serum. One gastrocnemius was collected, weighed, snap‐frozen in liquid nitrogen, and stored at −80°C for subsequent immunoblot analysis. The other gastrocnemius was collected and frozen in liquid nitrogen‐cooled isopentane for subsequent immunohistochemistry analysis. Freshly dissected quadriceps were collected and placed in ice‐cold mitochondrial isolation buffer (Frisard et al., 2015) for mitochondrial respiration and reactive oxygen species (ROS) measurements. For insulin signaling data collection, gastrocnemius muscle tissue was collected 10 min after insulin injection (1 U/kg).

### 2.5 Serum insulin levels

Fasting serum insulin levels were measured using an Insulin ELISA Kit (Thermo Fisher Scientific) according to the manufacturer's protocol.

### 2.6 Mitochondrial respiration

Seahorse XFp (Agilent Technologies) Analyzer was used to determine mitochondria respiratory rates by measuring oxygen consumption rates (OCR). Mitochondria were isolated from freshly dissected quadriceps muscle, as previously described (Frisard et al., 2015). After mitochondria isolation, protein concentration was determined using a Pierce BCA protein assay kit (Thermo Fisher Scientific). Isolated mitochondria were then used to determine mitochondrial respiration rates using a Seahorse XFp Extracellular Flux Analyzer (Agilent Technologies), as previously described (Boutagy et al., 2015). Briefly, immediately after protein quantification, isolated mitochondria were plated on the Seahorse plate at a concentration of 3.5 μg/well in the presence of 10 mM pyruvate and 5 mM malate. Seahorse XFp Extracellular Flux Analyzer plate was then centrifuged at 2000 g for 20 min at 4°C. Pre‐warmed pyruvate/malate substrate solution was then added to each well, so volume per well was 180 μl. ADP (5 mM), oligomycin (2 μM), carbonyl cyanide‐4 phenylhydrazone (FCCP, 4 μM), and antimycin (4 μM) were successively injected to measure OCR for different respiratory states. All data were analyzed using the Agilent Seahorse Wave software.

In human myotubes, mitochondrial respiration was measure by exchanging cell media to XF Assay Medium containing modified DMEM supplemented with 2 mM glutamine and 5 mM glucose and placed in a non‐CO_2_ 37°C incubator for 30 min prior to the start of the assay. Different respiratory states were measured by successive injections of oligomycin (2 μM), FCCP (0.75 μM), and rotenone/antimycin A (0.5 μM). All data were analyzed using Agilent Seahorse Wave software, and values were normalized to protein concentration for each well.

### 2.7 Reactive oxygen species

H_2_O_2_ levels in skeletal muscle lysates were measured using an Amplex Red Hydrogen Peroxide assay (Thermo Fisher Scientific) according to the manufacturer's instructions. Briefly, fresh quadriceps muscle was homogenized in an ice‐cold homogenization buffer (50 mM Tris, 1% Triton X‐100, 150 nM NaCl) and centrifuged at 1000 g for 10 min at 4°C. The supernatant was collected and used to determine H_2_O_2_ levels. Fluorescence was measured by a microplate reader (Thermo Fisher Scientific) with excitation wavelength 530–560 nm and emission wavelength at 590 nm. Data were normalized to the homogenate protein concentration.

Myotubes grown on 96‐well plates were washed in pre‐warmed PBS, incubated with 1 μM CM‐H_2_DCFDA (Thermo Fisher Scientific) in non‐phenol red DMEM at 37°C for 30 min with 5% CO_2_, and washed again with PBS. Fluorescence was measured by a microplate reader (Thermo Fisher Scientific, Waltham, MA) with an excitation wavelength at 490 nm and an emission wavelength at 520 nm. Values were normalized to protein concentration for each well.

### 2.8 Glucose uptake

Differentiated myotubes were serum‐starved in serum‐free DMEM for 3 h. Myotubes were then treated with or without 100 nM of insulin for 1 h. Glucose uptake assay was then performed using an assay kit provided by Cayman Chemical, following the manufacture's protocol.

### 2.9 Immunohistochemistry

Cryosection (8 μM) of the gastrocnemius was cut using a Leica Cryostat (Leica Biosystems) at −20°C. Three cryosections from two groups were mounted onto the same glass microscope slide (Fisher Scientific) to reduce the variation in staining intensity between sections. Slides were fixed for 10 min in cold acetone. The following slides were rinsed 3 × 5 min in phosphate‐buffered saline (PBS), then permeabilized in 0.25% Triton X‐100 for 10 min. Subsequently, slides were rewashed with PBS, after which the sections were blocked in 5% BSA for 20 min and then exchanged with 5% BSA with Fab fragment goat anti‐mouse IgG (Jackson ImmunoResearch) for 30 min. Sections were then incubated overnight in Drp1 primary antibody (Cell Signaling) at 4°C. The slides were rewashed with 1% BSA in PBS, and sections were incubated in a second primary antibody COX IV (Cell Signaling) for 1 h. Following a further wash in 1% BSA in PBS, secondary Alexa Fluor fluorescent‐conjugated antibodies (Jackson ImmunoResearch) were applied for 1 h. After incubation, sections were washed in 1% BSA in PBS. During the second wash, DAPI was added and incubated for 5 min. After DAPI incubation, sections were washed once, and coverslips were mounted using Vectashield antifade mounting medium (Vector Laboratories). Negative controls were included where the primary antibody was omitted in order to confirm the absence of nonspecific staining.

### 2.10 Fluorescence microscopy

To measure fluorescence intensity, images of Drp1 and COX IV were captured using a Zeiss confocal microscopy (Carl Zeiss AG). In order to visualize Alexa Fluor 488, an argon laser was used to excite Alexa Fluor 488, and signaling was collected in the 492–537 nm range. Alexa Fluor 594 was excited by an argon laser (590 nm), and its emission was collected at 619 nm. All images were imaged using a Plan‐Apochromat 63x/1.40NA oil immersion objective. Exposure was adjusted slightly to maximize signal and avoid saturation. A total of seven images were taken per animal. Each contained an average of ~4 fibers, leading to a total of 28 fibers per animal analyzed for colocalization.

Colocalization was analyzed using the Zen Black 2.1 Colocalization (Carl Zeiss AG). The overlay quantification threshold was set by setting the background cut‐off values with single color channels. Weighted colocalization coefficients were used to represent the sum of the intensity of colocalizing pixels in channel 1 and 2 compared to the overall sum of pixel intensities above the threshold. The values could be 0 (no colocalization) or 1 (all pixels colocalize). The colocalization coefficient represents the weight colocalization coefficient of Drp1 (Green) with respect to COX IV (Red). Each muscle fiber was then analyzed individually to avoid variations in background noise between images.

### 2.11 Immunocytochemistry and quantification of mitochondrial morphology

Immunocytochemistry and quantification of mitochondrial morphology were performed as previously described (Kugler et al., 2020). Briefly, myotubes cultured on a 35 mm Collagen‐I coated glass‐bottom dish were stained with 100 nM concentration of MitoTracker^TM^ RedFM (Thermo Fisher Scientific) diluted in differentiation media for 15 min. A Zeiss confocal microscopy was then used to image the myotubes mitochondrial network using a 64x1.4NA oil objective. Fifteen images per repeat were taken for quantification.

Images were analyzed using the mitochondrial network analysis macro (MiNA) tool developed for use on FIJI distribution of ImageJ as previously described (Valente et al., 2017). Images of mitochondria morphology were used to measure individual non‐networked mitochondria, the number of mitochondrial networks, branch length per network, and the number of branches per network. Data for the number of individual non‐networked mitochondria and the number of mitochondrial networks from each image were normalized by total MitoTracker intensity per nucleus.

### 2.12 Immunoblot analyses

Gastrocnemius muscles were mechanically homogenized in ice‐cold homogenization buffer supplemented with protease and phosphatase inhibitors and centrifuged at 12,000 rpm for 15 min at 4°C. Cell lysates were homogenized, as previously described (Kugler et al., 2020). Protein concentrations from myotubes, gastrocnemius, and mitochondria fraction were determined by Pierce BCA Protein Assay Kit (Thermo Fisher Scientific), and equal amounts of protein were subjected to SDS‐Page using 4%–20% gradient polyacrylamide gels (Bio‐Rad) and transferred to a nitrocellulose membrane. Membranes were probed with antibodies recognizing phosphorylated Drp1 Ser^616^ (cat# 3455), phosphorylated Drp1 Ser^637^(cat# 6319), Drp1 (cat# 8570), Opa1 (cat# 67589), Voltage‐dependent anion channel (VDAC, cat# 4661), GAPHD (cat# 2118), phosphorylated Akt Ser^473^ (cat# 9271), Akt (cat# 9272; Cell Signaling, Danvers, MA), Fis1 (cat # sc‐376447), BECN1 (cat# sc‐48341), MFF (cat# sc‐398617; Santa Cruz Biotechnology, Dallas, TX), OXPHOS Cocktail (human cat# ab110411, rodent cat# ab110413), Mfn1 (cat# ab104274), Mfn2 (cat# ab56889), P62 (cat# ab56416), Parkin (cat# ab77924), Pgc1‐α (cat# ab106814; Abcam, Cambridge, MA), and Mid51 (cat# 20164–1‐AP; proteintech). Membranes were probed with an IRDye secondary antibody (Li‐Cor) and quantified using Odyssey CLx software (Li‐Cor). Data were normalized to GAPDH protein expression for whole‐tissue lysates and VDAC for the mitochondrial fraction.

### 2.13 Statistical analysis

Group data are expressed as mean ± SEM. Group by time interaction for body weight, GTT, and ITT was analyzed using repeat measures ANOVA. Comparisons of subject characteristics were performed using an independent *t*‐test. Comparisons of insulin signaling data were performed using two‐way ANOVA followed by LSD multiple comparisons test. Comparisons of other parameters were performed using one‐way ANOVA followed by LSD multiple comparisons test. Pearson correlation analysis was used to assess linear relationships. All calculations were performed with SPSS statistical software (27.0; SPSS, Inc). All tests were two‐tailed, with a statistical significance set at *p* < 0.05.

## 3 RESULTS

### 3.1 Pharmacological inhibition of Drp1 improved glucose tolerance in diet‐induced obese mice

We first assessed mouse metabolic characteristics (Table 1). HFD‐fed mice had a significantly higher body weight when compared to LFD‐fed mice (*p* < 0.05). However, HFD‐fed mice with 1‐week of Mdivi‐1 treatment had significantly lower body weight when compared to saline‐treated HFD‐fed mice (Table1, *p* < 0.05). There were no differences in food consumption or gastrocnemius muscle mass between groups. Fasting blood glucose levels were significantly elevated in HFD‐fed mice regardless of Mdivi‐1 treatment (*p* < 0.05, Table 1). Interestingly, the HFD group had significant increases in fasting insulin, HOMA‐IR, and AUC of blood glucose levels in the GTT test when compared to the LFD (Table 1; Figure 1a,b, *p* ≤ 0.05), but these increases were not observed (fasting insulin and HOMA‐IR) or significantly reduced (AUC of blood glucose in GTT) in the HFD+Mdivi‐1 group. Regarding the ITT test, there is a trend toward significance between groups (Figure 1c, *p* = 0.086). Further statistical analysis revealed that blood glucose levels were significantly elevated at 30, 60, and 90 min during ITT test in the HFD group when compared to the LFD group; however, these elevations were significantly reduced in the HFD+Mdivi‐1 group at time points 30 and 60 min (Figure 1c, *p* < 0.05,). There were no differences in the inverse AUC of blood glucose levels in the ITT test between groups (Figure 1d).

**TABLE 1:** Animal characteristics

	LFD	HFD	HFD+Mdivi‐1
Body weight before injection (g)	25.3 ± 0.33	28.9 ± 0.26*	27.81 ± 0.63*
Final body weight (g)	26.7 ± 0.42	30.0 ± 0.34*	28.1 ± 0.52*, #
Food consumption per day (g)	3.71 ± 0.12	3.68 ± 0.15	3.54 ± 0.05
Gastrocnemius weight (mg)/body weight (g)	5.7 ± 0.2	5.5 ± 0.4	5.3 ± 0.2
Fasting glucose before injection (mg/dl)	171.8 ± 7.38	204.3 ± 10.52*	205.9.6 ± 7.74*
Fasting glucose (mg/dl)	193.6 ± 4.05	233.3 ± 9.77*	232.8 ± 10.36*
Fasting insulin (μIU/ml)	10.34 ± 1.78	25.84 ± 7.56*	17.61 ± 3.71
HOMA‐IR	4.86 ± 0.77	14.46 ± 4.04*	9.80 ± 2.12

Note

Data are presented as mean ± SEM.

Abbreviation: HOMA‐IR, Homeostatic Model Assessment of Insulin Resistance (fasting insulin concentration[μIU/ml) × fasting glucose concentration [mg/dl]/405).

* *p* < 0.05 versus LFD.

# *p* < 0.05 versus HFD.

**FIGURE 1:**
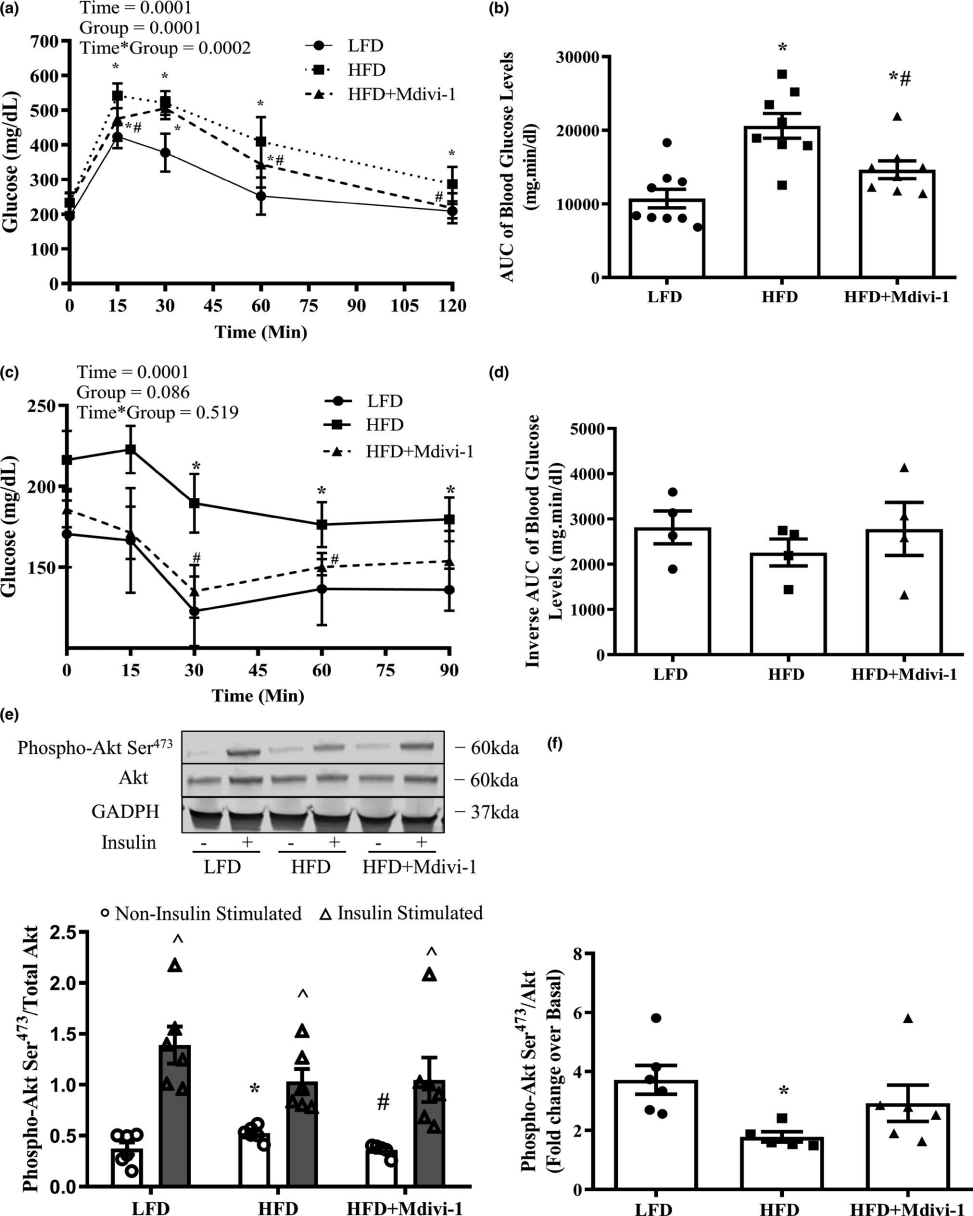
GTT, ITT, and skeletal muscle insulin signaling in mice fed either a LFD or HFD with or without Mdivi‐1 treatment. (a) Blood glucose levels during GTT test; (b) Blood glucose area under the curve (AUC) during GTT test; (c) Blood glucose levels during ITT test; (d) Inverse glucose area under the curve (AUC) during ITT test; (e) Phosphorylation of Akt Ser^473^/Akt under Non‐insulin stimulated and Insulin‐stimulated conditions; (f) Fold change in Akt Ser^473^ phosphorylation/total Akt under Insulin‐stimulated condition over Non‐insulin stimulated condition. Data are presented as mean ± SEM. *n* = 4–9/group. *p < 0.05 versus LFD, ^#^ *p* < 0.05 versus HFD, ^*p* < 0.05 versus respective Non‐insulin stimulated condition

### 3.2 Pharmacological inhibition of Drp1 improved skeletal muscle insulin signaling in diet‐induced obese mice

Basal Akt Ser^473^ phosphorylation was elevated (28.5%) in mice fed a HFD when compared to LFD (Figure 1e, *p* < 0.05), but was normalized with Mdivi‐1 treatment (Figure 1e, *p* < 0.05). The phosphorylation of Akt Ser^473^ was significantly elevated under insulin‐stimulated conditions compared to the basal condition in all three groups. However, the fold change (ratio of insulin‐stimulated over basal values) in the phosphorylation of Akt Ser^473^ in skeletal muscle was significantly reduced by 108% in mice fed a HFD in comparison to LFD counterparts (Figure 1f, *p* < 0.05), but was increased by 70% in mice treated with Mdivi‐1, and displayed virtually no difference when compared to the LFD group (Figure 1f).

### 3.3 Mdivi‐1 treatment reduced Drp1 translocation to mitochondria in skeletal muscle from diet‐induced obese mice

**FIGURE 2:**
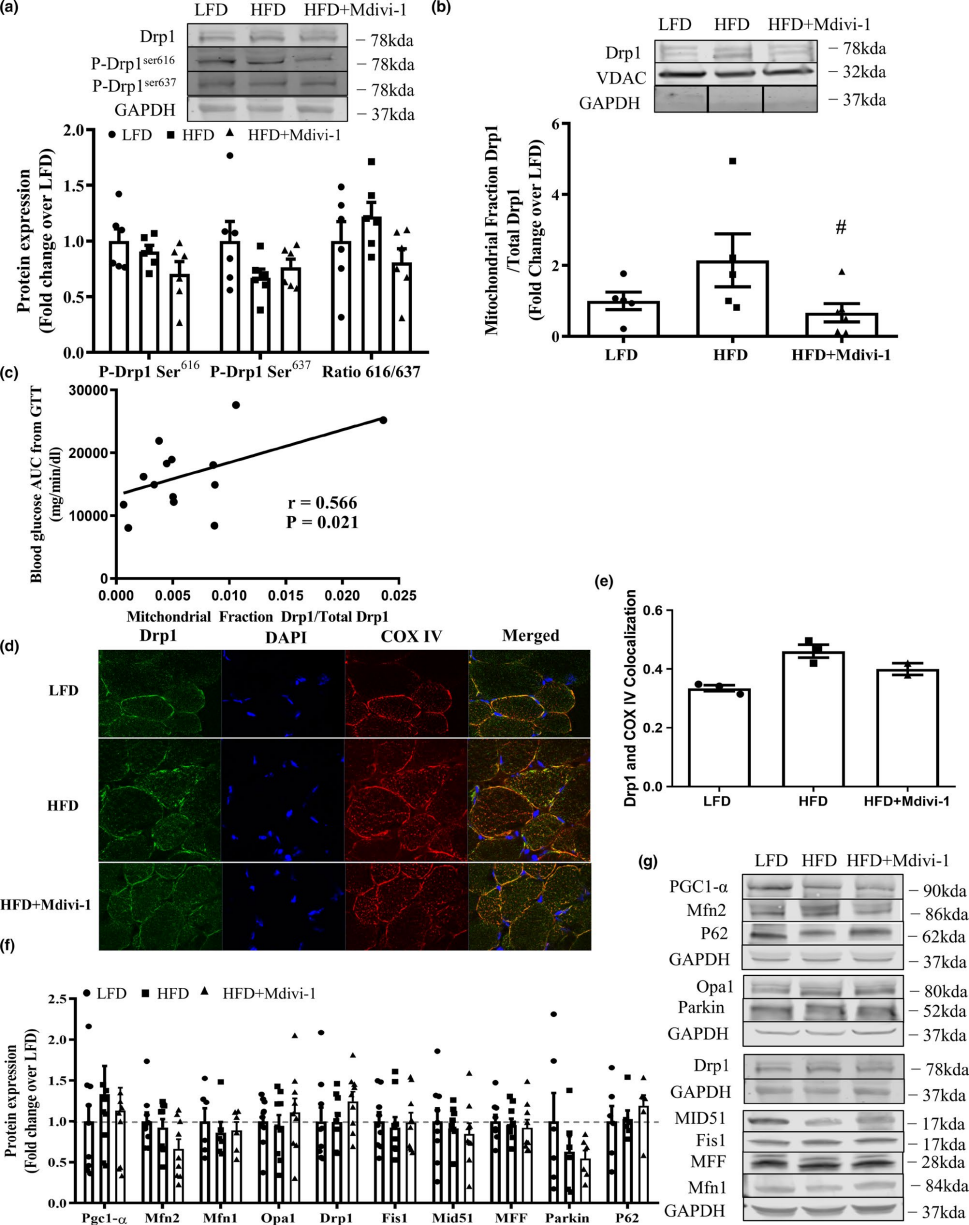
Drp1 activity and protein expression of mitochondrial quality control markers in skeletal muscle from mice either fed a LFD or HFD with or without Mdivi‐1 treatment. (a) Drp1 phosphorylations; (b) Ratio of Drp1 in the mitochondria fraction and whole tissue Drp1; (c) Correlation with the Drp1 translocation to mitochondria and blood glucose area under the curve (AUC) during GTT test (*r* = 0.566, *p* = 0.021). (d) Representative images of Drp1 colocalization with the mitochondrial marker COX IV in a section of skeletal muscle; (e) Quantification of Drp1 colocalization with the mitochondrial marker COX IV (*n* = 2–3); (f) Expression of regulatory proteins related to mitochondrial quality control; (g) Representative immunoblots for (e). Data are presented as mean ± SEM. *n* = 5–9/group. ^#^ *p* < 0.05 versus HFD

We next examined the Drp1 phosphorylations and translocation to the mitochondria in skeletal muscle. Although not significant, the ratio of Drp1 Ser^616^ and Ser^637^ phosphorylation, a marker of Drp1 activity, had a trend toward a significant increase (22%) in mice fed a HFD in comparison to the LFD counterparts (Figure 2a, *p* = 0.06). This elevation was attenuated in HFD‐fed mice treated with Mdivi‐1 (Figure 2a, *p* = 0.058*)*. The Mdivi‐1 treatment further reduced the translocation of Drp1 to the mitochondria (ratio of Drp1 mitochondria fraction to whole tissue Drp1) by 68.9% in HFD‐fed mice (Figure 2b, *p* < 0.05). In addition, the level of Drp1 translocation to mitochondria was positively associated with AUC of blood glucose levels in the GTT test (Figure 2c, *r* = 0.566, *p* = 0.021). We further examined Drp1 translocation to mitochondria using immunohistochemistry. Colocalization analysis of Drp1 and COX IV (mitochondrial marker) revealed that ~37% more mitochondria colocalized with Drp1 in skeletal muscle from the HFD‐fed mice than the LFD‐fed mice, whereas the HFD+Mdivi‐1 had a ~13% reduction in colocalization in comparison to the HFD (Figure 2d,e).

Neither 5 weeks of HFD feeding nor 1‐week of Mdivi‐1 treatment altered the expression of any other protein markers of mitochondrial biogenesis, fission, fusion, or mitophagy/autophagy (Figure 2f,g).

### 3.4 Pharmacological inhibition of Drp1 attenuated H_2_O_2_ content in skeletal muscle from diet‐induced obese mice

**FIGURE 3:**
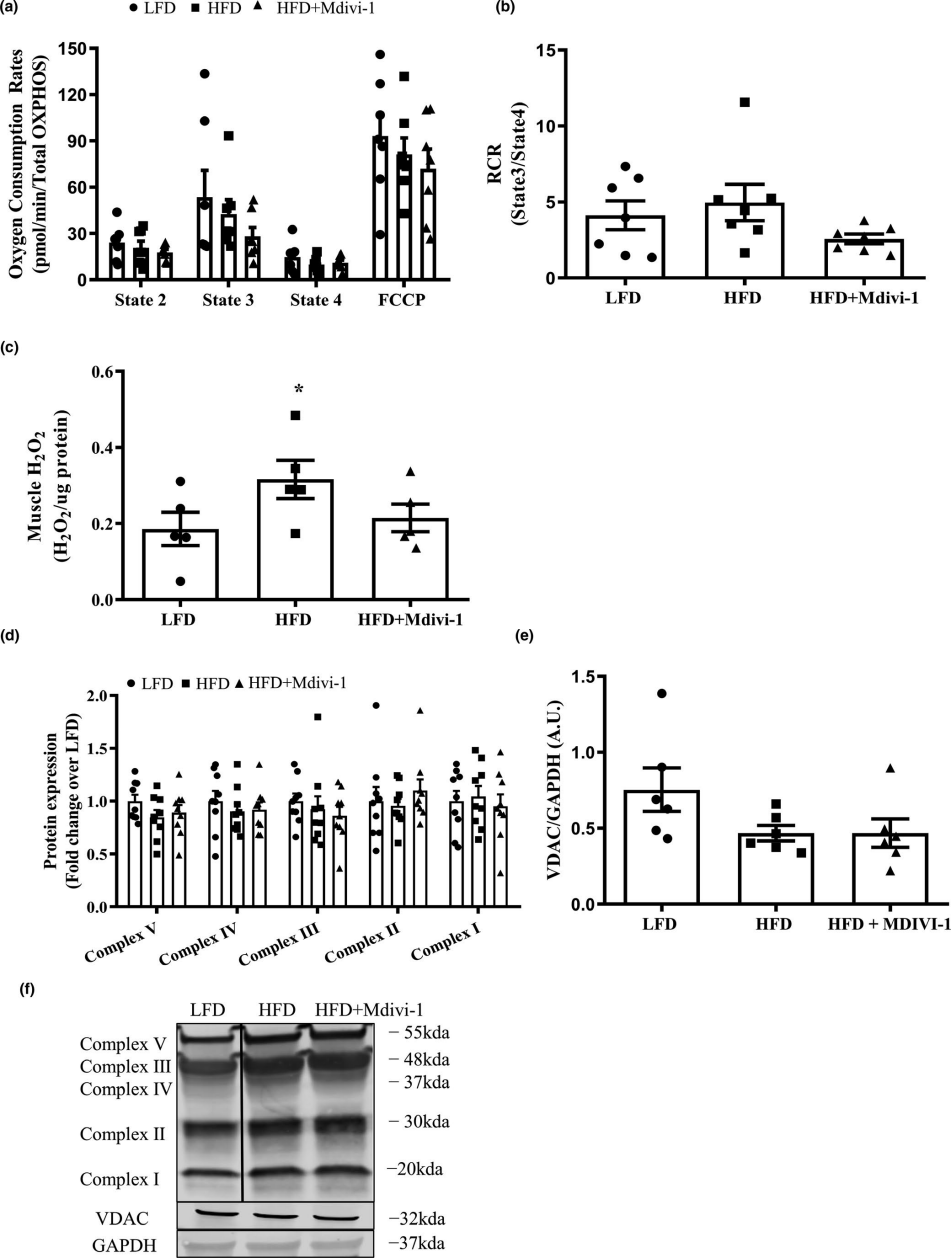
Mitochondrial function and content in skeletal muscle from mice fed either a LFD or HFD with or without Mdivi‐1 treatment. (a) Mitochondrial oxygen consumption rates under different respiration states; (b) Respiratory control ratio (RCR, State3/State 4); (c) H_2_O_2_ content in muscle homogenates; (d) OXPHOS protein content; (e) VDAC protein content; (f) Representative immunoblots for (d) and (e). Data are presented as mean ± SEM. *n* = 5–9/group. *p < 0.05 versus LFD

No difference was found in mitochondrial oxygen consumption rates (OCR) under different states of respiration between groups (Figure 3a,b). Interestingly, there was a significant increase (41.2%) in H_2_O_2_ content in HFD‐fed mice when compared to LFD‐fed mice (Figure 3c, *p* = 0.05) and such increase was attenuated in HFD‐fed mice treated with Mdivi‐1. There were no differences between groups in protein expressions of OXPHOS complexes and VDAC (Figure 3d–f).

### 3.5 Mdivi‐1 treatment reduces mitochondrial fragmentation in myotubes from obese insulin‐resistant humans

Subject characteristics are presented in Table 2. We selected severely obese subjects who exhibited an insulin‐resistant phenotype with significantly elevated fasting insulin levels and HOMA‐IR than the lean insulin‐sensitive controls (*p* < 0.05).

**TABLE 2:** Human subject characteristics

	Lean	Obese
Age	26.9 ± 4.1	37.5 ± 3.4
Weight (kg)	61.1 ± 2.8	137.4 ± 7.8*
BMI (kg/m^2^)	22.1 ± 1.23	49.5 ± 2.51*
Fasting glucose (mg/dl)	85.8 ± 2.06	91.2 ± 2.57
Fasting insulin (uLu/ml)	6.0 ± 0.82	16.0 ± 1.18*
HOMA‐IR	1.3 ± 0.17	3.6 ± 0.28*

Note

Data are presented as mean ± SEM

Abbreviations: BMI, body mass index; HOMA‐IR, Homeostatic Model Assessment of Insulin Resistance (fasting insulin concentration[μIU/ml) × fasting glucose concentration [mg/dl]/405).

* *p* < 0.05 versus Lean.

Myotubes derived from obese insulin‐resistant subjects were treated with different concentrations of Mdivi‐1, and images of the mitochondrial network morphology were taken (Figure S1A). It was determined that myotubes treated with a 20 μM concentration of Mdivi‐1 was the lowest concentration that showed the most considerable reduction in the volume of individual non‐networked and number of mitochondrial networks when compared to untreated myotubes (Figure S1B‐D). Therefore, myotubes derived from obese humans were treated with a 20 μM concentration of Mdivi‐1 for the remainder of the study.

**FIGURE 4:**
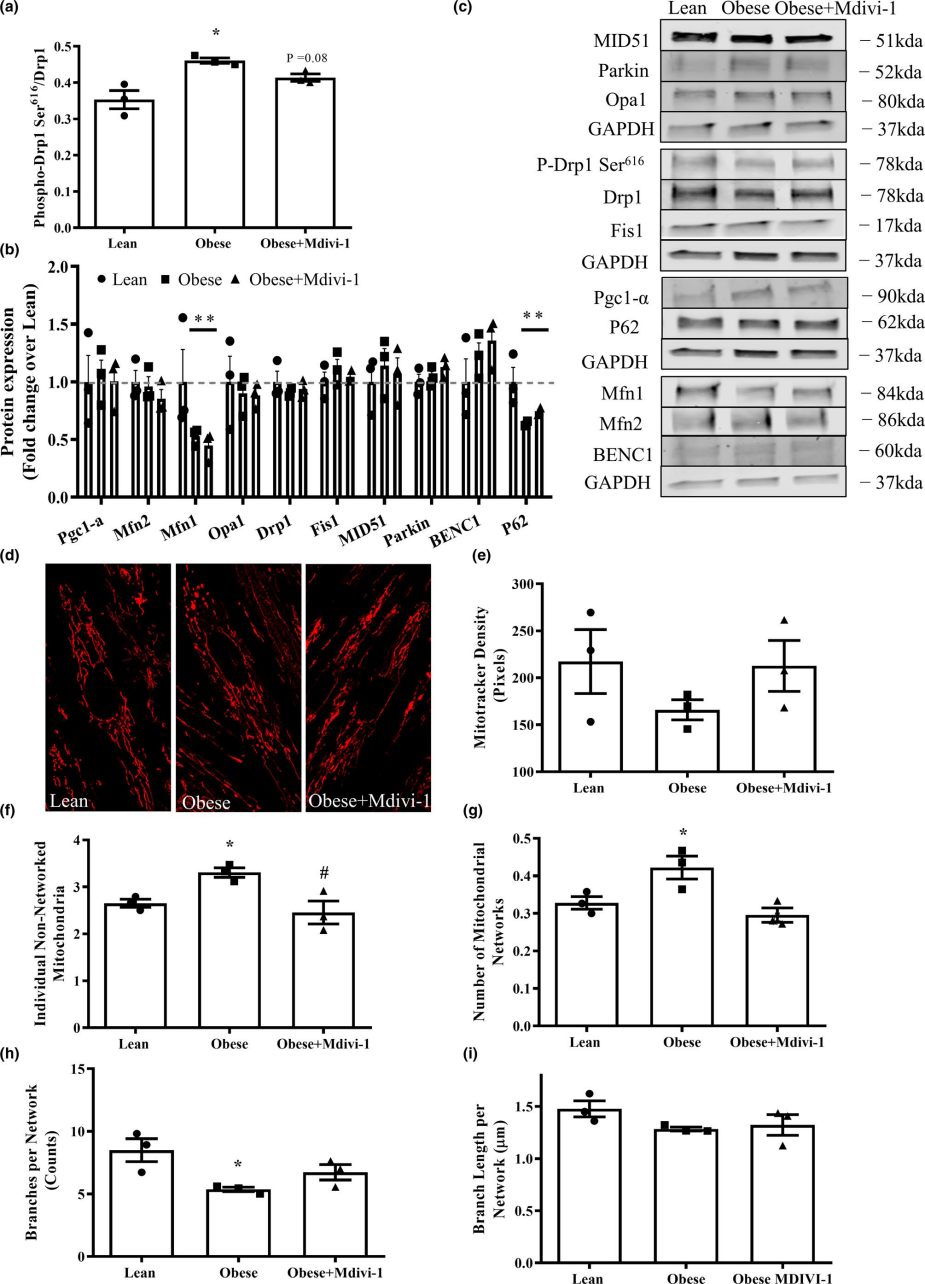
Protein expression of mitochondrial quality control markers and mitochondrial network morphology in myotubes derived from lean, insulin‐sensitive and obese, insulin‐resistant humans with or without Mdivi‐1 treatment. (a) Ratio of phosphorylated Drp1 Ser^616^ over Drp1; (b) Expression of regulatory proteins related to mitochondrial quality control; (c) Representative immunoblots in (a) and (b); (d) Representative images of myotubes stained with MitoTracker^TM^ RedFM; (e) MitoTracker intensity per nucleus; (f) Number of individual non‐networked mitochondria; (g) Number of mitochondrial networks; (h) Average number of branches per network (network size); (i) Average branch length per network (network size). Data are presented as mean ± SEM. *n* = 3 independent experiments of HSkMCs pooled from six obese insulin‐resistant humans. *p < 0.05 versus Lean; ^#^ *p* < 0.05 versus Obese; *p* = 0.08 versus Obese

We next evaluated the effects of Mdivi‐1 on Drp1 and other regulatory proteins of mitochondrial quality control in myotubes from lean insulin‐sensitive and obese insulin‐resistant humans. Myotubes from obese insulin‐resistant humans had a significant increase of 30.5% in the phosphorylation of Drp1 Ser^616^ in comparison to lean insulin‐sensitive counterparts (Figure 4a, *p* < 0.05). As expected, Mdivi‐1 treatment (12 h) tended to reduce the phosphorylation of Drp1 Ser^616^ (10.2%, *p* = 0.08). Additionally, protein expressions of mitochondrial fusion marker Mfn1 and autophagy marker P62 were significantly reduced in myotubes from obese insulin‐resistant individuals when compared to the lean insulin‐sensitive controls, regardless of Mdivi‐1 treatment (Figure 4b, *p* < 0.05). Mdivi‐1 treatment also did not alter the protein expression of any other markers of mitochondrial biogenesis, fission adaptors, and mitophagy between groups (Figure 4b,c).

Regarding mitochondrial morphology, myotubes derived from obese insulin‐resistant individuals exhibited greater mitochondrial fragmentation (Figure 4d) with significantly higher numbers of non‐networked individual mitochondria and mitochondrial networks (Figure 4f,g, *p* < 0.05), and smaller mitochondrial network sizes (Figure 4h, *p* < 0.05) when compared to myotubes from lean insulin‐sensitive humans. However, Mdivi‐1 treatment improved the mitochondrial network morphology with a significant reduction in the numbers of non‐networked individual mitochondria and mitochondrial networks when compared to vehicle‐treated myotubes (Figure 4f,g, *p* < 0.05). Mitochondrial density shown as MitoTracker intensity per nucleus (Figure 4e) and average branch length per network (Figure 4i) were not statistically different between groups.

### 3.6 Mdivi‐1 improves insulin action in myotubes from obese insulin‐resistant humans

**FIGURE 5:**
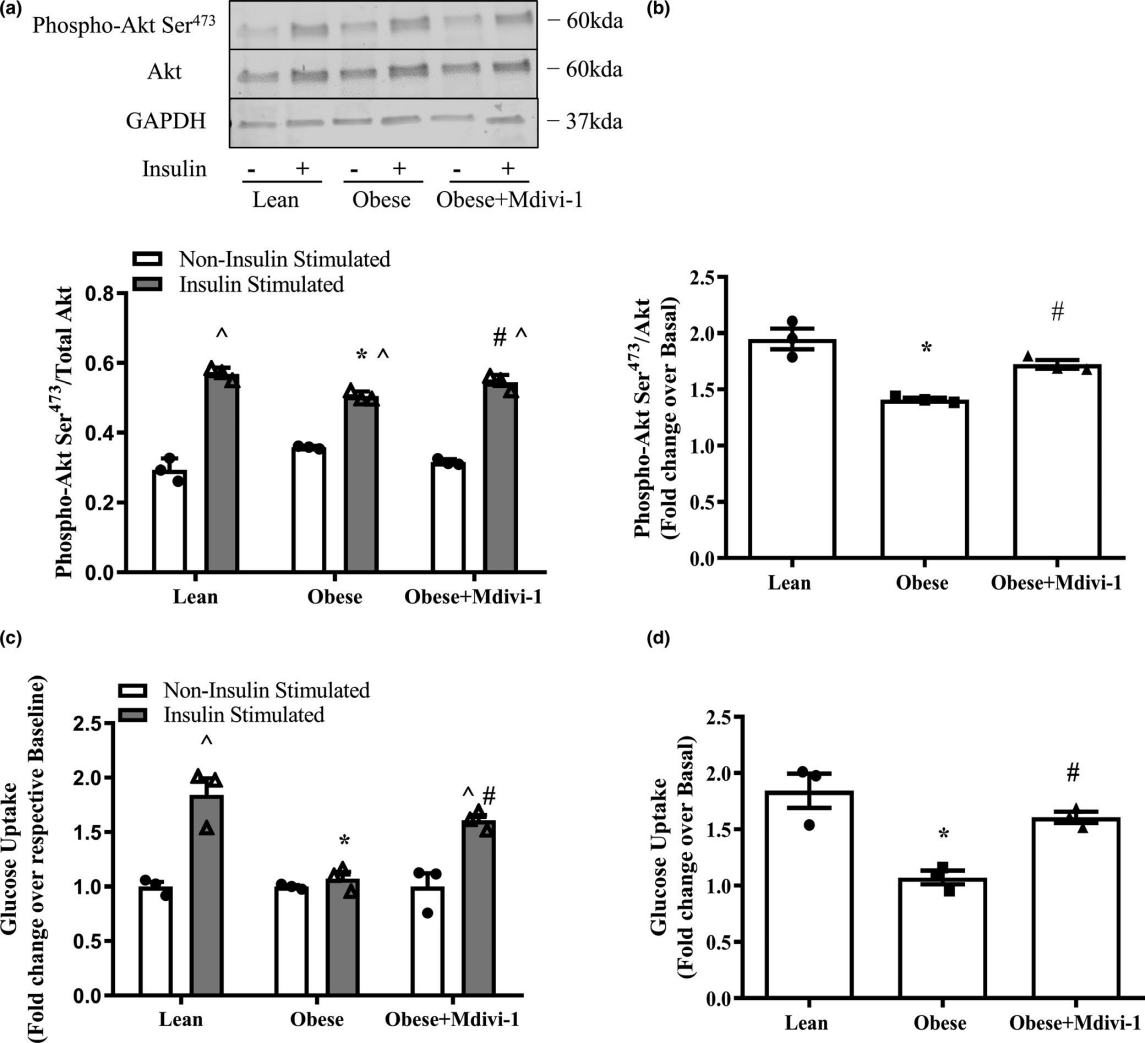
Indices of insulin action in myotube derived from lean, insulin‐sensitive and obese, insulin‐resistant humans with or without Mdivi‐1 treatment. (a) Akt Ser^473^ phosphorylation/total Akt under basal and insulin‐stimulated conditions; (b) Fold change in Akt Ser^473^ phosphorylation/Total Akt under insulin‐stimulated condition over basal condition; (c) Glucose uptake under Non‐insulin stimulated and Insulin‐stimulated conditions; (d) Fold change in glucose uptake under Insulin‐stimulated condition over Non‐insulin stimulated condition. Data are presented as mean ± SEM. *n* = 3 independent experiments of HSkMCs pooled from 6 obese insulin‐resistant humans. *p < 0.05 versus Lean; ^#^ *p* < 0.05 versus Obese; ^*p* < 0.05 versus respective Non‐insulin stimulated condition

Myotubes from obese insulin‐resistant humans had significant reductions in insulin‐stimulated phosphorylation of Akt Ser^473^ and glucose uptake in comparison to lean insulin‐sensitive counterparts, which were restored by Mdivi‐1 treatment (Figure 5a,c, *p* < 0.05). Similarly, the ratio of insulin‐stimulated phosphorylation of Akt Ser^473^ and glucose uptake over the basal state, surrogate markers of insulin action, were both significantly improved in myotubes from obese insulin‐resistant subjects with Mdivi‐1 treatment when compared to the vehicle‐treated counterparts, and had virtually no differences when compared to the levels in myotubes from lean, insulin‐sensitive humans (Figure 5b,d, *p* < 0.05).

### 3.7 Mdivi‐1 treatment does not alter mitochondrial respiratory function but reduces ROS production in myotubes derived from obese insulin‐resistant humans

**FIGURE 6:**
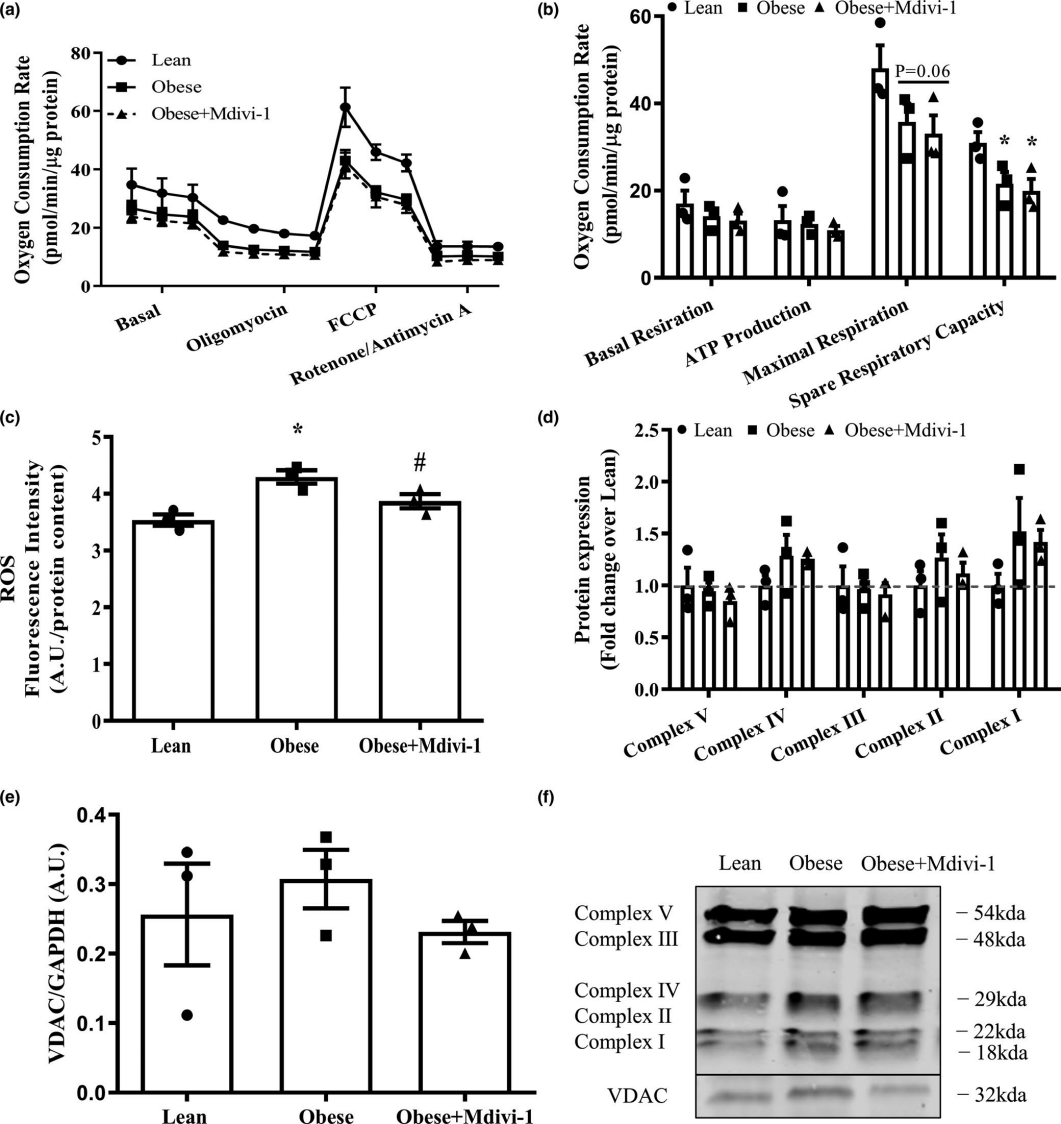
Mitochondrial function in myotubes derived from lean, insulin‐sensitive and obese, insulin‐resistant humans with or without Mdivi‐1 treatment. (a) Representative graph of oxygen consumption rates (OCR); (b) Oxygen Consumption Rates (OCR); (c) Intracellular reactive oxygen species (ROS) levels; (d) OXPHOS protein content; (e) VDAC protein expression; (f) Representative immunoblots for (d) and (e). Loading control was presented in Figure 4c. Data are presented as mean ± SEM. *n* = 3 independent experiments of HSkMCs pooled from six obese insulin‐resistant humans. *p < 0.05 versus Lean; ^#^ *p* < 0.05 versus Obese; *p* = 0.06 versus Lean

No differences in basal respiration and ATP production were observed between groups (Figure 6a,b). However, myotubes from obese insulin‐resistant subjects displayed a significant reduction in spare respiratory capacity (Figure 6b, *p* < 0.05) and a trend toward a significant reduction of maximal respiration (Figure 6b, *p* = 0.06) when compared to lean insulin‐sensitive counterparts, which were not rescued by Mdivi‐1 treatment. Interestingly, myotubes from obese insulin‐resistant subjects had a higher ROS production in than the lean insulin‐sensitive counterparts, which were effectively reduced with Mdivi‐1 treatment (Figure 6c, *p* < 0.05). There were no changes in the protein expressions of VDAC or any mitochondrial OXPHOS complex (Figure 6d–f).

## 4 DISCUSSION

In the development of obesity‐induced insulin resistance, mounting evidence indicates that excessive Drp1‐mediated mitochondrial fission is negatively correlated with skeletal muscle insulin action and whole‐body insulin sensitivity (Fealy et al., 2014; Jheng et al., 2012; Kugler et al., 2020). Herein, we demonstrated that Mdivi‐1, a pharmacological inhibitor targeting Drp1‐mediated mitochondrial fission, is efficacious in alleviating skeletal muscle insulin resistance and improving whole‐body glucose tolerance in the setting of obesity‐induced insulin resistance. In addition, these improvements appear to be, at least in part, due to mitochondrial H_2_O_2_ content. Our study adds novel knowledge from a translational perspective that targeting Drp1‐mediated mitochondrial fission in vivo could be a practical approach in alleviating skeletal muscle insulin resistance and enhancing whole‐body metabolic health in obesity.

The primary finding of our study was that Drp1 inhibition *in vivo* using the pharmacological inhibitor Mdivi‐1 attenuated skeletal muscle insulin resistance at the early stage in the development of diet‐induced obesity. The beneficial effects of Drp1 inhibition are in line with previous work, including ours that demonstrated the inverse relationship between Drp1 activity and skeletal muscle insulin sensitivity (Fealy et al., 2014; Kugler et al., 2020), as well as a study showing that single Mdivi‐1 injection transiently (i.e., 1 h prior to sacrifice) enhanced insulin signaling in a genetic model of obesity (Jheng et al., 2012). Our results extended these findings by demonstrating the short‐term efficacy of Drp1 inhibitor administration *in vivo* in the setting of obesity‐induced insulin resistance. Furthermore, it is worth mentioning that while the role of Drp1‐mediated mitochondrial fission in skeletal muscle insulin resistance is supported in the literature, it is almost exclusively based on findings from animal and C_2_C_12_ cell culture models. Consistent with previous findings, by utilizing a translational HSkMC model, our study demonstrated that inhibiting Drp1‐mediated mitochondrial‐fission also improved insulin action in human skeletal muscle. To our knowledge, this is the first study that demonstrated the beneficial effects of Drp1 inhibitor in human skeletal muscle, adding the translational potential of therapeutic strategies targeting Drp1‐mediated mitochondrial fission to treat insulin resistance and T2D.

Elevated Drp1(Ser^616^) phosphorylation and/or Drp1 protein content in skeletal muscle have been reported under obesity and insulin‐resistant conditions (Gundersen et al., 2020; Jheng et al., 2012; Leduc‐Gaudet et al., 2018; Liu et al., 2014). To our surprise, we did not see alterations in either of these two markers in the HFD group. The discrepancy is likely due to differences in methodology. For example, our study used a short‐term HFD feeding (5 weeks) and focused on the onset of obesity and insulin resistance, whereas other studies used a much longer duration of HFD intervention (>10 weeks; Jheng et al., 2012; Liu et al., 2014). Second, our study assessed these protein markers in gastrocnemius muscle, which is different from the other study in which increased Drp1 content was found in soleus muscle (Leduc‐Gaudet et al., 2018). In fact, when the EDL muscle was used, they did not find any change in Drp1 content following HFD feeding. Lastly, exccesive Drp1(Ser^616^) phosphorylation has been found in severely obese humans (Fealy et al., 2014; Gundersen et al., 2020; Kugler et al., 2020), suggesting there might be different Drp1 phosphorylation sites determining Drp1 activity between humans and rodents. Given that both Ser^616^ and Ser^637^ phosphorylation sites are important in determining Drp1 activity (Westermann, 2010), we evaluated these two phosphorylation site and found that there is a trend toward a significant increase in Drp1 Ser^616^/Ser^637^ ratio (*p* = 0.06), suggesting Drp1 activity is elevated in HFD group. Taken together, it suggests that different phosphorylation sites may determine Drp1 activity in the development of obesity and insulin resistance and future studies should consider to more comprehensively assess its activity when studying Drp1‐mediated mitochondrial fission in skeletal muscle.

The results from this study revealed that skeletal muscle H_2_O_2_ content was elevated following 5 weeks of HFD feeding, which was prevented by the inhibition of Drp1‐mediated mitochondrial fission. Our results are in line with previous work showing that inhibiting Drp1‐mediated mitochondrial fission leads to reduced ROS and improved insulin signaling (Fan et al., 2010; Jheng et al., 2012; Lin et al., 2018; Wu et al., 2011; Yu et al., 2008), but extends these findings by demonstrating a reduction of a specific form of ROS. H_2_O_2_ is converted from superoxide that leaks from the mitochondria and is a significant contributor to ROS production in skeletal muscle (Powers et al., 2011). While we did not directly measure mitochondrial H_2_O_2_ production in this study, Anderson et al. reported that 6 weeks of a HFD (similar duration to our study) markedly increased skeletal muscle mitochondrial H_2_O_2_ production and induced insulin resistance (Anderson et al., 2009). Therefore, it is plausible that Drp1 inhibition by Mdivi‐1 may rebalance the regulatory machinery of mitochondrial dynamics leading to reduced mitochondrial H_2_O_2_ production and subsequently improve insulin signaling in skeletal muscle from diet‐induced obese mice. Alternatively, Drp1 inhibition by Mdivi‐1 may induce improved antioxidant capacity, causing reduced H_2_O_2_ content in muscle tissue.

Although not a primary objective of this study, we found that a short‐term (5‐weeks) HFD did not alter mitochondrial respiration in skeletal muscle when NADH‐generating substrates were used. These findings are in agreement with several previous studies using rodent models (Hoeks et al., 2011; Leduc‐Gaudet et al., 2018). For example, animals fed a HFD for up to 8 weeks presented no reduction in skeletal muscle mitochondrial respiration despite the presence of insulin resistance (Bonnard et al., 2008). In contrast, Boudina and colleagues reported that 5 weeks of HFD feeding enhanced mitochondrial respiration in C57BL/6J mice using substrates directed at β‐oxidation (e.g., palmitoyl‐l‐carnitine; Boudina et al., 2012), suggesting a short‐term HFD may enhance fatty acid‐driven, but not NADH‐driven electron‐transfer pathway into the mitochondrial electron transport chain. Regarding Drp1‐mediated mitochondrial fission, Mdivi‐1 did not alter mitochondrial respiration in both myotubes from insulin‐resistant humans and isolated mitochondria from HFD‐fed mice, suggesting that Drp1‐mediated mitochondrial fission may not be a key regulator of NADH‐driven electron transfer pathway. Future research is warranted to investigate fatty‐acid oxidation and fatty‐acid‐driven mitochondrial respiration to determine whether Drp1‐mediated mitochondrial fission regulates these processes in skeletal muscle under the setting of obesity and insulin resistance. It is worth noting that the selection of the model to assess mitochondrial respiration (i.e., isolated mitochondria vs. permeabilized muscle fibers) may also play a role in the discrepancy of the findings as isolated mitochondria may only include a specific sub‐sarcolemmal mitochondria.

Recent studies reported several detrimental effects of inhibiting Drp1 in skeletal muscle by either genetic (e.g., Drp1 KO or KD) or pharmacological approaches (e.g., Mdivi‐1), including impaired insulin signaling, reduced mitochondrial respiration, and muscle atrophy (Dulac et al., 2020; Favaro et al., 2019; Ribas et al., 2016). These adverse effects of inhibiting Drp1 are in disagreement with our study. We found improved insulin signaling and did not observe any reduction in muscle weight or myotube size. These divergent findings may be due to (1) different animal models used and (2) the degree and duration to which Drp1 content/activity is reduced. Recent reports by Favaro et al., and Dulac et al., genetically reduced Drp1 in lean healthy mice, which caused imbalanced mitochondrial dynamics resulting in muscle atrophy (Dulac et al., 2020; Favaro et al., 2019). In contrast, this study focuses on obesity and insulin‐resistant conditions, which are accompanied by excessive Drp1 activity (e.g., Drp1 phosphorylation). Drp1 inhibition under these conditions likely rebalanced the regulatory machinery of mitochondrial dynamics. Altogether, it suggests that the balance in mitochondrial dynamics is essential for the regulation of skeletal muscle quality and function (Ferree & Shirihai, 2012). Second, our study used a pharmacological inhibitor that inhibited Drp1 translocation without total Drp1 content reduction in mice. In contrast, previous studies reported severe muscle atrophy induced by a significant reduction in skeletal muscle Drp1 content (>40%; Dulac et al., 2020; Favaro et al., 2019). This suggests that the extent to which Drp1 is reduced may be critical in regulating skeletal muscle quality and function. Interestingly, Moore et al. reported no muscle atrophy despite the fact that Drp1 was reduced to a comparable degree in female mice (Moore et al., 2019), suggesting there may also be sex differences in the role of Drp1 in regulating skeletal muscle quality and function. Future studies are warranted to (i) utilize Drp1 genetic deletion models to study skeletal muscle quality and function under diet‐induced obese and insulin‐resistant conditions; (ii) study sex difference upon Drp1 inhibition.

One interesting finding of this study is the partially prevented body weight gain in HFD‐fed mice treated with Mdivi‐1. Given the fact that there was no change in food consumption between groups, it suggests that Mdivi‐1 administration may enhance energy expenditure. A recent study showed that mice lacking Drp1 in the liver exhibited increased energy expenditure and protected against HFD‐induced obesity due to elevated expression of FGF21 (Wang et al., 2015). Therefore, Mdivi‐1 administration may have inhibited Drp1 in the liver leading to increased FGF21 expression and energy expenditure.

Mdivi‐1 is by far the most accessible pharmacologic inhibitor of the mitochondrial fission protein Drp1 (Cassidy‐Stone et al., 2008a). A large body of literature has shown that Mdivi‐1 selectively inhibits Drp1 in mammalian cells by binding at an allosteric site and suppressing Drp1 translocation to mitochondria (Smith & Gallo, 2017). Our Drp1 phosphorylation data, coupled with mitochondrial network structure data in vitro, demonstrated that Mdivi‐1 is an effective inhibitor of Drp1 and mitochondrial fission in skeletal muscle cells. Our findings are consistent with a previous study that utilized C_2_C_12_ muscle cell culture (Jheng et al., 2012), but in contradictory to another study (Bordt et al., 2017), which reported that Mdivi‐1 treatment did not inhibit Drp1 activity and improve mitochondrial elongation, suggesting Mdivi‐1 is not a specific Drp1 inhibitor. The discrepancy in results from different studies can be attributed to different protocols of Mdivi‐1 treatment (e.g., concentration, duration, and cell lines). Bordt et al., treated fibroblasts for 1–5 h with a concentration between 10 and 50 μM, whereas in our study, we treated human skeletal muscle cells for a much longer time (12 h) with 20 μM. Interestingly, Jheng et al., treated C_2_C_12_ cells for only 1 h but found Mdivi‐1 inhibited Drp1 phosphorylation and improved mitochondrial morphology, suggesting Mdivi‐1 may exert different effects in different types of cells.

Regarding the inhibitory effects of Mdivi‐1 on Drp1 in vivo, while we did not directly assess mitochondrial fission and network structure in vivo, we found that Drp1 content in mitochondria fraction and colocalizaion of Drp1 and Cox IV (mitochondria marker) data were reduced in mice treated with Mdivi‐1 for a week, suggesting Mdivi‐1 is effective in inhibiting Drp1 activity. Suprisingly, we did not observe any change in Drp1 Ser616 phosphorylation or Drp1 content in skeletal muscle tissue collected from mice treated with Mdivi‐1, which is in contrast to two studies investigating the effects of Mdivi‐1 injection on skeletal muscle in vivo (Giovarelli et al., 2020; Jheng et al., 2012). It should be noted that Jheng et al., utilized a higher dosage (44 mg/kg BW) and assessed the acute effect of Mdivi‐1 on Drp1 (1‐h post last injection). The other study conducted by Giovarelli and colleagues injected Mdivi‐1 for 8 weeks in their study. Taken together, it suggests that it may need a longer duration of Mdivi‐1 treatment in order to alter baseline Drp1 phosphorylation. Indeed, we found a trend toward a significant reduction in the ratio of Drp1 phosphorylations Ser^616/^Ser^637^ (an indicator of Drp1 activity). Future studies with a longer duration of Mdivi‐1 treatment should be warranted to validate the findings from this study. Overall, current studies focusing on the effects of Mdivi‐1 in skeletal muscle are scarce. Although our study extended previous findings and provided new evidence on the effects of in vivo Mdivi‐1 administration on mitochondrial integrity and insulin sensitivity, comprehensive evaluations of Mdivi‐1 with various dosages and duration on skeletal muscle mitochondrial dynamics, morphology, function, and metabolism are much needed in future studies. Our study has some limitations. First, we performed a systemic administration of Mdivi‐1. Therefore, we cannot rule out the contribution of other metabolic tissues (e.g., liver, pancreas, and fat) in improved whole‐body glucose tolerance following Mdivi‐1 treatment. Second, our study inhibited Drp1 for only one week. Although the long‐term efficacy and safety of Mdivi‐1 administration (i.e., 8‐weeks) have been validated in diabetic mice (Ayanga et al., 2016), it has yet to be studied in the context of skeletal muscle quality (e.g., muscle mass, fiber size) and metabolic function and should be warranted in future studies.

In conclusion, we provide evidence that in vivo inhibition of Drp1 using Mdivi‐1 alleviated impairment in skeletal muscle insulin signaling and improved whole‐body glucose tolerance in diet‐induced obese mice. Follow‐up analyses determined that these improvements appear to be, at least in part, due to reduced H_2_O_2_ content in skeletal muscle. The results from this study also provide new information that inhibiting Drp1 is effective in improving skeletal muscle insulin sensitivity in human skeletal muscle. Together, our study adds the translational potential of targeting Drp1‐mediated mitochondrial fission as a viable approach to treat obesity‐induced insulin resistance and type 2 diabetes.

## CONFLICT OF INTEREST

The authors have no conflicts of interest, financial or otherwise, to declare.

## AUTHOR CONTRIBUTIONS

K.Z. and B.A.K. did the conception and design of the research; J.A.H provided HSkMCs; B.A.K., W. D., A.L.D., J.P.G., M.C.A., P.D.N., and K.Z., performed the experiments; B.A.K. and K.Z. analyzed the data; B.A.K and K.Z. interpreted the results of the experiments, prepared the Figures, and drafted the manuscript; B.A.K and K.Z. edited and revised the manuscript; all authors approved the final version of the manuscript.

## Supporting information



Supplementery Material

Supplementery Material
