# Supplementary figures and images for: Pharmacological inhibition of dynamin‐related protein 1 attenuates skeletal muscle insulin resistance in obesity
Source: Physiol Rep. 2021 Apr 27;9(7):e14808. doi: 10.14814/phy2.14808 (PMC8077121; doi:10.14814/phy2.14808)

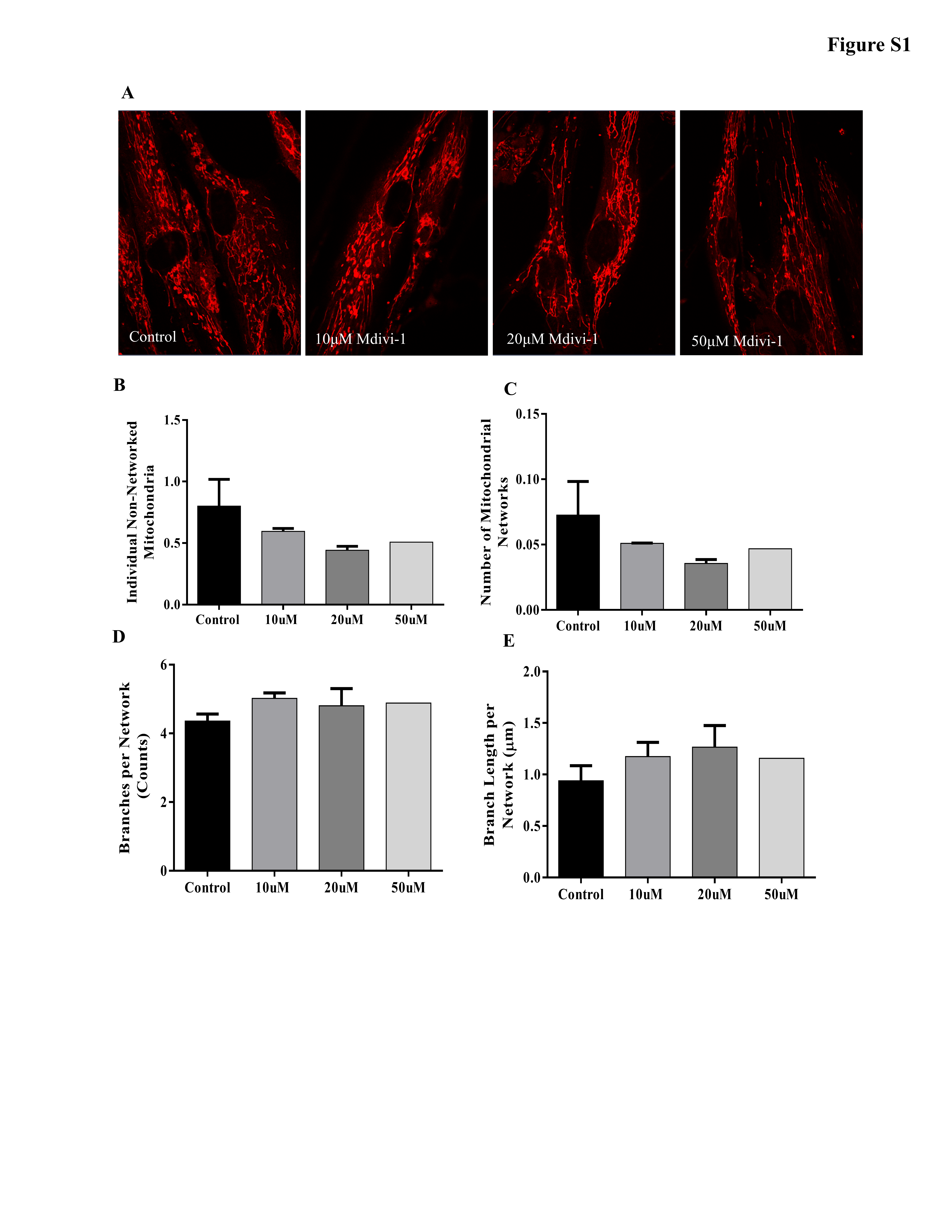

Supplement: Supplementary file 1 — Supplementery Material [file PHY2-9-e14808-s002.tif]
