# Supplementary material for: Pharmacological inhibition of dynamin‐related protein 1 attenuates skeletal muscle insulin resistance in obesity
Source: Physiol Rep. 2021 Apr 27;9(7):e14808. doi: 10.14814/phy2.14808 (PMC8077121; doi:10.14814/phy2.14808)
Supplement: Supplementary file 2 — Supplementery Material [file PHY2-9-e14808-s001.docx]

**Supplementary Fig. 1:** Mdivi-1 dose dependent change of mitochondria morphology in severely obese myotubes. **A:** Representative images of myotubes stained with MitoTracker^TM^ RedFM; **B:** Number of individual non-networked mitochondria; **C:** Number of mitochondrial networks; **D:** Average number of branches per network (network size); **E:** Average branch length per network (network size); Data are presented as Mean ± SEM.
